# Surface Display of Cholera Toxin B Subunit Recombinant *Escherichia coli* Ghosts Further Enhances Resistance to *Chlamydia abortus* Infection in Mice

**DOI:** 10.3390/microorganisms12081656

**Published:** 2024-08-13

**Authors:** Huaiyu Zhang, Yunhui Li, Wei Li, Zhaocai Li, Jizhang Zhou, Dewen Tong

**Affiliations:** 1 College of Veterinary Medicine, Northwest A&F University, Yangling 712100, China; 2 State Key Laboratory for Animal Disease Control and Prevention, College of Veterinary Medicine, Lanzhou University, Lanzhou Veterinary Research Institute, Chinese Academy of Agricultural Sciences, Lanzhou 730000, China; 3 Gansu Province Research Center for Basic Disciplines of Pathogen Biology, Lanzhou 730046, China

**Keywords:** *Chlamydia abortus*, bacterial ghost, vaccine, CTB, mucosal immunity

## Abstract

*Chlamydia abortus* (*C. abortus*) is an important zoonotic pathogen that seriously endangers the development of animal husbandry. Vaccination is the most effective approach to preventing *C. abortus* infection. We previously reported a recombinant *Escherichia coli* ghost (rECG)-based *C. abortus* vaccine that demonstrated outstanding protective efficacy. In this study, we further attempted to fuse the cholera toxin B subunit (CTB), a widely studied potent mucosal immune adjuvant, with macrophage infectivity potentiator (MIP), a candidate antigen of *C. abortus*, on the surface of the rECG and explore its protective effect against *C. abortus* infection. The MIP fusion protein was highly expressed in the rECGs, and the CTB-modified rECGs significantly induced the activation of mouse bone marrow-derived dendritic cells in vitro. Intranasal immunization with rECGs induced a Th1-biased cellular immune response. Compared to the rECGs without CTB, the CTB-modified rECGs induced higher concentrations of IgA in the serum and vaginal wash solution. Moreover, in a mouse infection model, the CTB-modified rECGs significantly improved the clearance efficiency of *C. abortus* and reduced the pathological damage to the uterus. This study demonstrates that incorporating CTB into rECGs significantly enhances the immunogenic potential of the rECG vaccine and can significantly enhance its protective efficacy against a *C. abortus* challenge.

## 1. Introduction

*Chlamydia abortus* (*C. abortus*) is an obligate intracellular parasitic bacterium with a unique biphasic developmental growth cycle [1]. Moreover, it can cause enzootic abortion in ewes, characterized by abortion, stillbirth, or mummified fetuses in primiparous animals [2]. Although antibiotics can be used for treatment, infected animals are often asymptomatic, resulting in untimely or incomplete treatment and drug resistance [3]. In addition, the cross-species transmission of *C. abortus* poses hidden risks to public health [4]. Although many types of vaccines have been studied intensively for decades, a reliable vaccine has not yet been developed. The live attenuated *C. abortus* 1B vaccine, which is considered to prevent infection, carries a risk of virulence return [5], whereas inactivated vaccines are usually unable to prevent *Chlamydia* infection [6]. Therefore, new vaccine candidates are being constantly developed.

Bacterial ghosts (BGs) are empty bacterial shells formed by the loss of nucleic acids and other bacterial components due to physical, chemical, or biological factors [7]. BGs have unique adjuvant properties because they retain their natural cell surface structure. BGs are becoming novel delivery systems for vaccines, drugs, and active substances due to their unique spatial structure. We have previously demonstrated that a *C. abortus* subunit vaccine using recombinant *Escherichia coli* ghosts (rECGs) as carriers can significantly reduce infection with *C. abortus* [8]. In addition to the expression of heterologous antigens in BGs using genetic engineering technology, BGs can be modified via the fusion expression of adjuvant proteins (e.g., flagellin and fms-like tyrosine kinase 3-ligand) to enhance their adjuvant properties or increase their targeting [9,10].

*C. abortus* mainly infects animals through the nasal cavity or gastrointestinal mucosa [11]. The clearance of *Chlamydia* in the genital tract is closely associated with IgG and IgA, and significant protection against *Chlamydia* is associated with T-helper type-1 (Th1) cellular immune responses and high levels of interferon-gamma (IFN-γ) [12]. Therefore, an ideal *C. abortus* vaccine candidate should contain components that can induce strong IgA and Th1-type immune responses. Cholera toxin (CT) is one of the strongest mucosal immune adjuvants discovered. However, its clinical application is limited owing to its toxicity [13]. The CT B subunit (CTB) removes the toxicity of CT while retaining its strong adjuvant activity. Any protein can be genetically linked to CTB to form a fusion protein, and the recombinant chimera induces protective immunity [12,14].

In this study, we attempted to use CTB to modify ECGs and to evaluate whether its immunoprotective effect could be enhanced through the mucosal immune pathway. We used the surface display vector lpp’-ompA [15], a chimera consisting of Braun’s lipoprotein (lpp) and the five transmembrane segments of outer membrane protein A (ompA), to fuse the expression of a protective antigen macrophage infectivity potentiator (MIP) [16] of *C. abortus* and CTB on the surface of *E. coli* and prepare the rECG. The immune response and immunoprotective efficacy of the rECGs were evaluated in a mouse model. These results strongly support the development of rECGs as vaccine candidates against *C. abortus*.

## 2. Materials and Methods

### 2.1. Animals, Cells, Plasmids, and Strains

Specific pathogen-free-grade, female, 6–8-week-old BALB/c mice were purchased from the Laboratory Animal Centre of Lanzhou Institute of Veterinary Medicine, and all animal experimental protocols were approved by the Animal Ethics Committee of Lanzhou Institute of Veterinary Medicine (No. LVRIAEC-2023-035).

The pACYCDuet-1 and pUC-ΔWK-E plasmids [8] and competent *E. coli* C43 (DE3) and *C. abortus* GN6 strains [17] were all stored at the Lanzhou Veterinary Research Institute in the Zoonosis Laboratory. L929 mouse fibroblasts (ATCC # CCL-1) were purchased from the cell bank of the Chinese Academy of Sciences (Shanghai, China). The CTB and lpp’-ompA sequences were described previously [15,18] and were synthesized by Hongxun Biotech, China. Unless otherwise stated, all *E. coli* strains were cultured in Luria–Bertani (LB) medium. Plasmid-carrying strains were cultured and propagated in LB containing kanamycin (100 μg/mL) or chloramphenicol (50 μg/mL). *C. abortus* GN6 strain was generated by propagation on L929 cells, as previously described, and stored at −80 °C.

### 2.2. Plasmid Construction and Induced Expression of Recombinant Protein

Table 1 lists all polymerase chain reaction (PCR) amplification primers used. The lpp’-ompA and MIP genes were amplified using the primers lpp-F/lpp-R and MIP-F/MIP-R, respectively, and the amplified products were sequentially inserted into multiple cloning site 1 (MCS1) of the pACYCDuet-1 plasmid using the restriction endonuclease method to generate the pA-lpp’-MIP plasmid. The CTB-F/CTB-R primers were then used to amplify the CTB sequence and inserted into the pA-lpp’-MIP plasmid to generate the pA-lpp’-MIP-CTB plasmid. The antigen expression plasmids were subsequently transformed into *E. coli* C43 (DE3) cells. Isopropyl-β-D-thiogalactopyranoside (IPTG) was added to the bacterial culture medium (0.1 mM) and the bacteria were cultured with shaking at 20 °C for 12 h to induce antigen expression.

### 2.3. Western Blot

Bacteria with induced expression were collected via centrifugation, dissolved in sodium dodecyl sulfate buffer, and heated at 100 °C for 10 min. The cleavage products were separated on 12% gels. Then, a mouse anti-MIP antibody (ABGENT; 1:3000) or mouse anti-CTB antibody (ABclone; 1:3000) was used to detect the corresponding protein, and the protein bands were imaged.

### 2.4. Flow Cytometry and Confocal Laser Scanning Microscopy Analyses

After induction, bacteria were recovered by centrifugation. The bacterial pellets were fixed in 5% glutaraldehyde for 30 min. Each sample was first incubated with mouse anti-MIP antibody (1:200 dilution), followed by Alexa Fluor 594-labeled anti-mouse IgG antibody (Abcam, Cambridge, UK) at 1:500 dilution. Finally, the bacterial genome was labeled with 4′,6-diamidino-2-phenylindole (DAPI, 1 μg/mL), and the fluorescence signal was detected by flow cytometry (Backman CytoFLEX LX, Brea, CA, USA) and confocal laser scanning microscopy (Leica TCS SP8, Wetzlar, HE, Germany).

### 2.5. Preparation and Antigen Quantification of rECGs

The rECGs were prepared according to a previously described method [8]. Briefly, a single bacterial clone cotransformed with an expression plasmid and lysis plasmid was selected and cultured. IPTG (0.1 mM) was added to the culture medium, and the cells were cultured at 20 °C for 12 h to induce MIP expression. The induced bacteria were diluted in fresh medium to an optical density at 600 nm (OD_600_) of approximately 0.4. We set the temperature to 42 °C for continued culture for 5 h to lyse the bacteria and form BGs. Finally, the bacterial precipitate was processed into a lyophilized powder, and whether it was completely inactivated was tested on solid LB medium supplemented with double antibodies (kanamycin and chloramphenicol).

As we have previously described, an enzyme-linked immunosorbent assay (ELISA) was used to quantify the MIP content in the rECGs [8]. A known concentration of purified rMIP protein was used as a reference, and the content of the fusion MIP in different rECGs was expressed as μg MIP/mg total lysate protein.

### 2.6. BMDC Activation Assay

Mouse bone marrow-derived dendritic cells (BMDCs) were cultured and induced in complete medium (Pricella, Wuhan, China) supplemented with GM-SCF (0.02 μg/L) and IL-4 (0.02 μg/L), as previously described [19]. BMDCs were seeded in 6-well plates (3 × 10^6^ cells/well) and treated with rMIP (1 μg/mL) or rECG (ghost–cell ratio of 5:1) for 18 h. The cells were collected and resuspended in 1% bovine serum albumin and then blocked with anti-mouse CD16/CD32 antibody (clone 2.4G2) for 30 min at 4 °C. Subsequently, the cells were incubated with phycoerythrin (PE)-labeled CD11c antibody (clone N418), allophycocyanin (APC)-labeled CD80 (clone 16-10A1), and fluorescein isothiocyanate (FITC)-labeled major histocompatibility complex II (MHC II) (clone GL1) at 4 °C in the dark for 0.5 h. After washing three times, 7-AAD reagent was added to exclude dead cells, and the fluorescence signal was detected by flow cytometry. The flow antibodies were from Thermo Fisher, Waltham, MA, USA. The culture supernatant was collected and the concentrations of IL-12p70, IL-6, and TNF-α were measured using ELISA kits.

### 2.7. Immunization and Challenge

Female BALB/c mice were randomly divided into four groups. In the two rECG vaccine groups, each mouse was intranasally immunized with 2 mg of rECG-lpp’-MIP or rECG-lpp’-MIP-CTB. Each mouse in the subunit vaccine group was intranasally immunized with 20 μg of rMIP prepared with Freund’s adjuvant. In the PBS group, each mouse was given the same dose of PBS. One milligram of rECG was equivalent to 2 × 10^9^ colony-forming units of bacteria. Three immunizations were administered at intervals of two weeks between each administration. Each mouse was injected subcutaneously with 2.5 mg of medroxyprogesterone one week before infection to synchronize the estrous cycle and promote productive infection. Three weeks after the last immunization, each mouse was challenged transcervically with the *C. abortus* GN6 strain (5 × 10^5^ inclusion body-forming units (IFU)). The course of infection was monitored by swabbing the vaginal vault with calcium alginate at selected intervals and counting the recoverable IFUs according to standard methods, as previously described [20].

### 2.8. Spleen Lymphocyte Proliferation Test

CCK-8 was used to measure the proliferation capacity of mouse spleen lymphocytes. Briefly, mice were euthanized by carbon dioxide inhalation and immersed in 75% ethanol for 5 min. The spleen was removed in a super clean bench, RPMI 1640 culture medium (Gibco, Grand Island, NY, USA) was added, and the spleen was ground into a cell suspension with sterile instruments and filtered through a 70 μm nylon filter membrane. Then, 90 μL of the cell suspension (10^6^ cells/mL) and 10 μL of recombinant MIP (10 μg/mL) were added to a 96-well cell culture plate and incubated for 72 h. Subsequently, 10 μL of CCK-8 reagent (Solarbio, Beijing, China) was added to each well and they were incubated for 4 h, after which the absorbance at 450 nm was detected using a microplate reader (TECAN infinite 200 Pro, Mannedorf, Switzerland).

### 2.9. Antibody and Cytokine Testing

Serum samples were collected from mice on days 0, 14, 28, 42, and 56 after the first immunization, or vaginal wash solution samples were collected on day 42, and the levels of MIP-specific antibodies were measured using ELISA. Briefly, microplates were coated with rMIP (2 μg/mL) in carbonate-bicarbonate buffer overnight at 4 °C. After washing, nonspecific binding was blocked using 1% bovine serum albumin. A serum or vaginal wash solution sample was added and the samples were incubated at 37 °C for 2 h. After washing, HRP-labeled goat anti-mouse IgA, IgG, IgG1, or IgG2a (Southern Biotech, Birmingham, AL, USA) was added and the samples were incubated at 37 °C for 2 h. After the substrate had formed a colored product, a microplate reader was used to read the OD value at 450 nm. 

According to the manufacturer’s instructions, the concentrations of IFN-γ, IL-12p70, TNF-α, IL-4, and IL-10 in the spleen lymphocyte culture supernatant were detected using an ELISA kit (eBioscience, San Diego, CA, USA). The concentrations of different cytokines in each sample were calculated based on a standard curve generated simultaneously.

### 2.10. Histopathology

Uterine tissue was subjected to hematoxylin and eosin (H&E) staining and histopathological analysis, as previously described [21]. Briefly, on day 10 after challenge, mice were euthanized through carbon dioxide inhalation. Dissection was performed under sterile conditions, and uterine samples were carefully isolated with surgical instruments, fixed with 4% paraformaldehyde, embedded in paraffin, sectioned, stained with H&E, and photographed for analysis.

### 2.11. Isolation of C. abortus from the Uterus

The collected uterine samples were prepared into uterine homogenates using a tissue homogenizer, filtered through a 70 μm nylon filter, and inoculated onto L929 cell monolayers in 10-fold serial dilutions to isolate *C. abortus*, as described previously [22]. Infection was assessed by counting *C. abortus* IFUs. The limit of detection was 4 × 10^2^ IFUs per uterus.

### 2.12. Statistical Analysis

All data were analyzed using the GraphPad Prism 10 software. The significance of the differences between different groups was determined using one-way ANOVA. A *p* value of <0.05 was considered to indicate statistical significance.

## 3. Results

### 3.1. Construction of Surface-Displayed Foreign Antigen Plasmids

**Figure 1:**
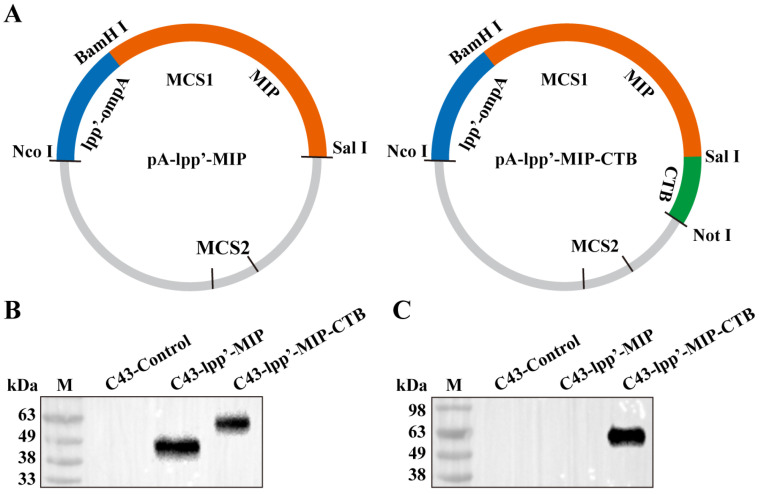
Construction of surface display plasmid and identification of fusion protein. (**A**) Schematic diagram of recombinant plasmids pA-lpp’-MIP and pA-lpp’-MIP-CTB. Fusion protein expression was detected by Western blotting using monoclonal antibodies against MIP (**B**) and monoclonal antibodies against CTB (**C**). M: protein marker.

As shown in Figure 1A, two plasmids consisting of the lpp’-ompA chimera fused with MIP or MIP-CTB were constructed. The pA-lpp’-MIP and pA-lpp’-MIP-CTB plasmids were transformed into *E. coli* C43(DE3) (C43-lpp’-MIP and C43-lpp’-MIP-CTB, respectively) and the expression of the fusion protein was induced. The expression of fused proteins lpp’-MIP and lpp’-MIP-CTB was analyzed by Western blotting, and the strain (C43-Control) transformed with the pACYCDuet-1 intact plasmid was used as a negative control. Corresponding bands were detected for both C43-lpp’-MIP and C43-lpp’-MIP-CTB using an MIP antibody (Figure 1B). The target band only appeared in the C43-lpp’-MIP-CTB band detected using the CTB antibody (Figure 1C).

### 3.2. Detection of Fusion Protein Surface Display

**Figure 2:**
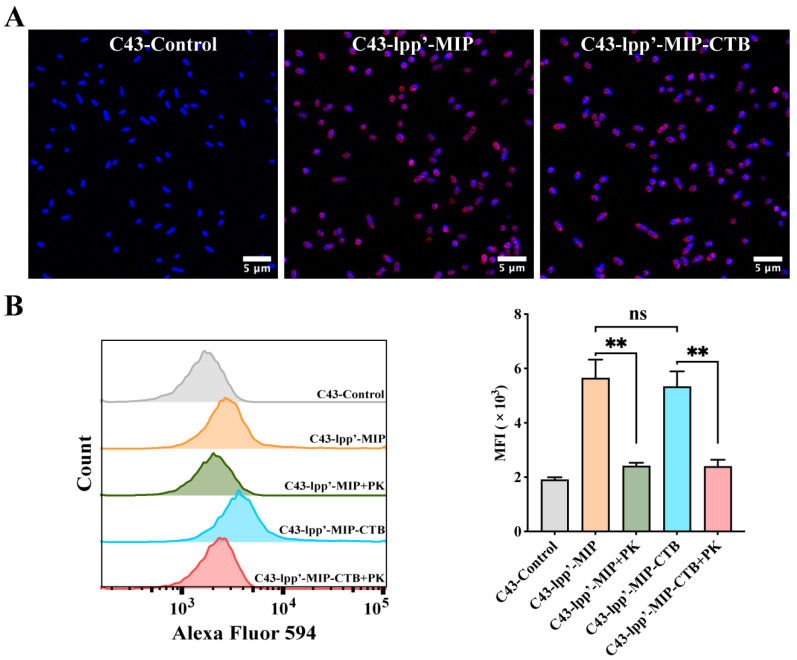
Analysis of display of MIP fusion proteins on bacterial surfaces. (**A**) MIP antibody was used to incubate bacteria, followed by Alexa Fluor 594-labeled anti-mouse IgG antibody (red) for indirect immunofluorescence analysis. Bacterial genomes were labeled with DAPI (blue). (**B**) Proteinase K-treated (+PK) or untreated C43-lpp’-MIP and C43-lpp’-MIP-CTB were incubated with MIP antibodies, followed by flow cytometry detection with Alexa Fluor 594-labeled anti-mouse IgG antibodies. Final data are shown as MFI. Results are expressed as mean ± standard deviation (SD). ** *p* < 0.01; ns, no significance.

MIP antibodies were incubated with bacteria with induced expression and observed by confocal microscopy to detect the display of fusion proteins on the bacterial surfaces. Red fluorescence signals were observed on the surfaces of C43-lpp’-MIP and C43-lpp’-MIP-CTB. In contrast, no fluorescence signal was observed in C43-Control (Figure 2A). Flow cytometry analysis showed that compared with those of C43-Control, the average fluorescence intensity (MFI) of C43-lpp’-MIP and C43 lpp’-MIP-CTB was significantly greater. The MFI was significantly reduced when the induced bacteria were pretreated with proteinase K (Figure 2B), indicating that the fusion protein displayed on the bacterial surface was partially digested and destroyed by protease K.

### 3.3. Preparation of rECGs

The quantification of the MIP in the rECGs was performed using ELISA. The levels of MIP fusion proteins in rECG-lpp’-MIP and rECG-lpp’-MIP-CTB were similar, reaching more than approximately 12 μg/mg (Table 2). In addition, no viable bacteria were detected in the lysed bacterial pellet after freeze-drying, indicating that all bacteria were completely inactivated.

### 3.4. Effect of rECGs on the Maturation and Activation of BMDCs

**Figure 3:**
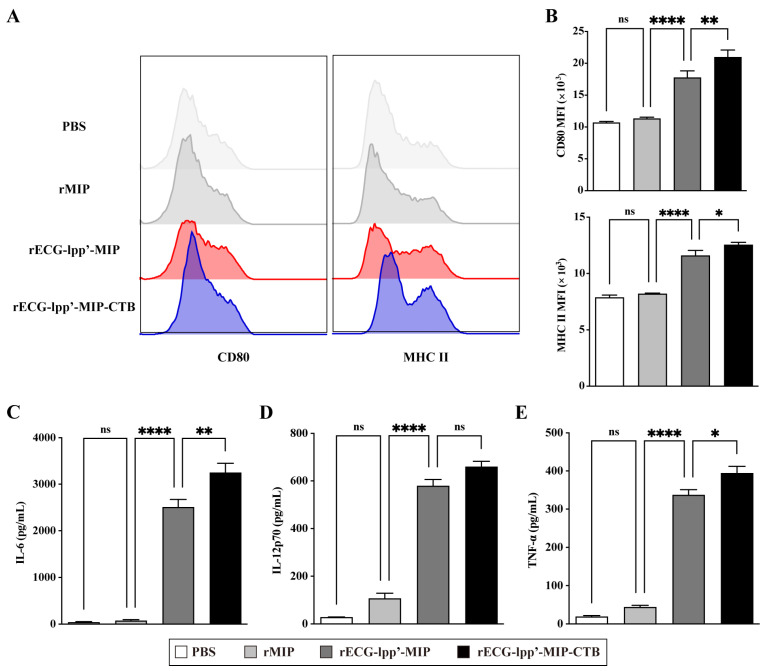
Effects of rECGs on BMDCs’ maturation and activation. (**A**,**B**) After BMDCs were stimulated by rECGs in vitro, the CD80 and MHC II expression frequencies of CD11c^+^ cells were detected by flow cytometry. The final data are shown as the MFI. Content of cytokines IL-6 (**C**), IL-12p70 (**D**), and TNF-α (**E**) in BMDC culture supernatants. Results are expressed as means ± SD. * *p* < 0.5, ** *p* < 0.01, **** *p* < 0.0001; ns, no significance.

DCs are the primary antigen-presenting cells, and their maturation and activation are crucial for antigen uptake, processing, and presentation. Therefore, we evaluated the effects of rECGs on the maturation and activation of BMDCs. Compared with the control (PBS treatment) group, the rECG treatment group significantly induced the expression of CD80 and MHC II, and the CD80 levels in the rECG-lpp’-MIP-CTB group were significantly higher than those in the rECG-lpp’-MIP (Figure 3A,B). In addition, both the rECG groups significantly induced the secretion of IL-6, IL-12p70, and TNF-α, and rECG-lpp’-MIP-CTB induced significantly higher IL-6 and TNF-α levels than did rECG-lpp’-MIP (Figure 3C,D).

### 3.5. rECGs Induce Specific Antibody Responses in Mice

**Figure 4:**
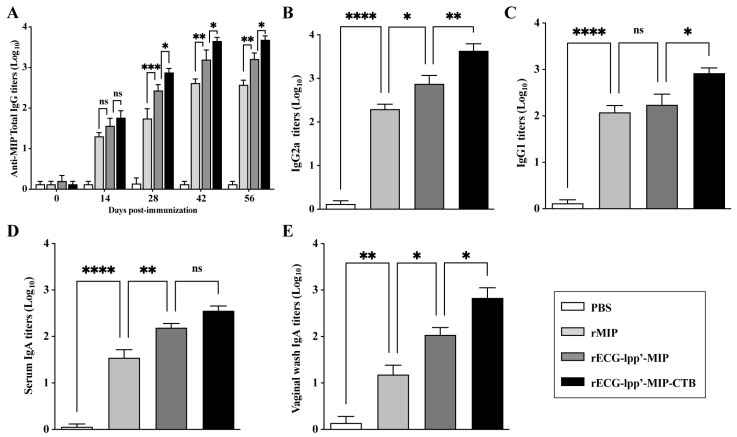
Humoral immune response induced by rECGs in mice. (**A**) Dynamics of MIP-specific IgG in serum samples collected 0, 14, 28, 42, and 56 days after the first immunization. Titers of MIP-specific IgG2a (**B**) and IgG1 (**C**) in serum samples 42 days after the first immunization. MIP-specific IgA titers in serum samples (**D**) and vaginal lavage fluid (**E**) 42 days after the first immunization. Results are expressed as mean ± SD. * *p* < 0.05, ** *p* < 0.01, *** *p* < 0.001, **** *p* < 0.0001; ns, no significance.

Given the strong adjuvant activity of rECGs modified with CTB, we evaluated the feasibility of using rECGs as a mucosal immune vaccine. As shown in Figure 4A, except for those in the PBS group, the serum total IgG titers increased significantly over time in all groups after primary, secondary, and tertiary immunization. They reached a maximum of 42 days after the first immunization. The titers of IgG2a and IgG1 induced in the rECG-lpp’-MIP-CTB group were significantly higher than those in the rECG-lpp’-MIP group 42 days after the first immunization (Figure 4B,C). As expected, the IgA titers in the serum and vaginal washes of the rECG-lpp’-MIP-CTB group 42 days after the first vaccination were significantly higher than those of the rECG-lpp’-MIP group (Figure 4D,E).

### 3.6. Cell-Mediated Immune Response

**Figure 5:**
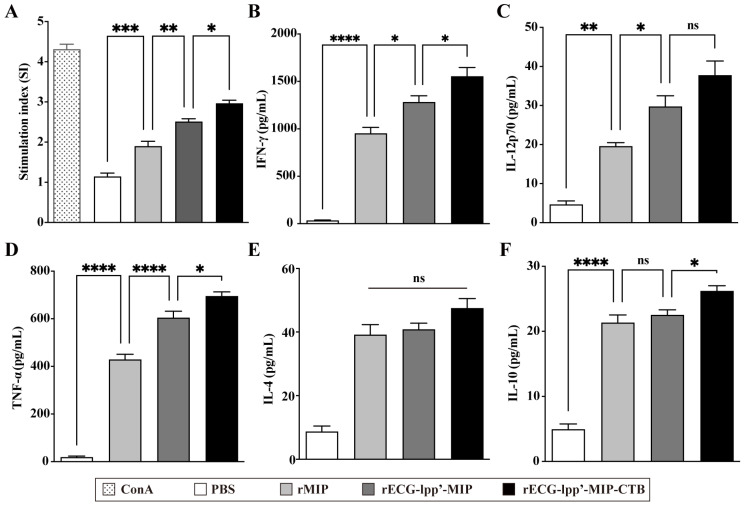
Cellular immune response induced by rECGs in mice. (**A**) Specific splenic lymphocyte proliferation response in immunized mice, with concanavalin A (ConA)-treated group serving as a positive control. Content of IFN-γ (**B**), IL-12p70 (**C**), TNF-α (**D**), IL-4 (**E**), and IL-10 (**F**) in lymphocyte culture supernatants. Results are expressed as mean ± SD. * *p* < 0.05, ** *p* < 0.01, *** *p* < 0.001, **** *p* < 0.0001; ns, no significance.

Spleen lymphocytes were isolated from mice 7 days after the last immunization. After restimulation with rMIPs, a CCK-8 assay was used to detect the proliferation of splenic lymphocytes. The stimulation index (SI) of the rECG-lpp’-MIP-CTB group was significantly higher than that of the rECG-lpp’-MIP group (Figure 5A). ELISA was used to detect Th1- and Th2-type cytokines in the culture supernatants. rECG-lpp’-MIP and rECG-lpp’-MIP-CTB induced high levels of IFN-γ and TNF-α and low levels of IL-12p70, IL-4, and IL-10. In addition, the levels of IFN-γ, TNF-α, and IL-10 were significantly higher in the rECG-lpp’-MIP-CTB group than in the rECG-lpp’-MIP group, except for IL-12p70 and IL-4 (Figure 5B–F).

### 3.7. Protective Efficacy of rECGs

**Figure 6:**
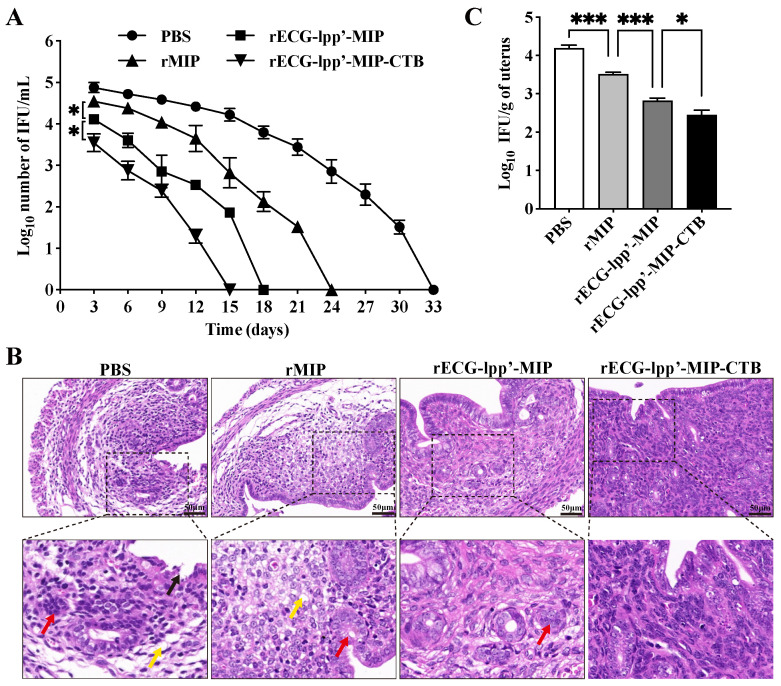
Protective effect of rECGs on mice infected with *C. abortus*. (**A**) Vaginal swabs were collected every 3 d post-challenge to enumerate the *C. abortus*-recoverable IFUs. The mean recoverable IFUs are expressed as log_10_ IFU/mL ± SD. (**B**) Histopathological analysis of uterine tissue sections. Lysis and necrosis of the lamina propria are indicated with black arrows, uterine gland cavity occlusion is indicated with red arrows, and lamina propria edema is indicated with yellow arrows. (**C**) Quantitative isolation of *C. abortus* in uterine homogenates of mice 7 days after challenge. * *p* < 0.05, *** *p* < 0.001.

Infection was assessed based on the shedding of *C. abortus* in the vaginas of the mice. Within 15 days after the challenge, the IFUs in the rECG-lpp’-MIP and rECG-lpp’-MIP-CTB groups were consistently lower than those in the PBS group. The clearance rate of rECG-lpp’-MIP-CTB was higher than that of rECG-lpp’-MIP, and no shedding of *C. abortus* was detected 15 days after the challenge, whereas the rECG-lpp’-MIP group took 18 days (Figure 6A).

Histopathological analysis was performed on uterine tissue sections from post-challenge mice. The PBS and rMIP groups exhibited the most severe uterine tissue damage, mainly manifested as endometrial epithelial shedding, edema and the dissolution of the lamina propria, a blurred uterine gland structure, and a reduced number of uterine glands. The uterine tissue damage in the rECG-lpp’-MIP-CTB group was mild, and epithelial cell watery degeneration and lymphocyte infiltration were occasionally observed (Figure 6B). The results of the uterine *C. abortus* burden test showed that the number of bacterial IFUs in the rECG vaccine group was significantly lower than that in the rMIP group. The number of bacterial IFUs in the rECG-lpp’-MIP-CTB group was significantly lower than that in the rECG-lpp’-MIP group (Figure 6C).

## 4. Discussion

*C. abortus* seriously harms global livestock herds and poses potential threats to human life and health. In addition, owing to the backward production technology and poor immune protection effects of traditional vaccines, there is an urgent need to develop a reliable, effective, new vaccine with few side effects to protect animals from abortive *C. abortus* infections and eliminate the public health safety threats. Eko et al. developed a series of *Chlamydia* vaccines using *Vibrio cholerae* ghosts (rVCGs) as delivery systems. rVCGs can induce strong Th1-type and high-level humoral immune responses, even without the addition of adjuvants. At the same time, rVCGs can generate immune memory and resist reinfection by *Chlamydia* [23,24,25]. BGs are promising *Chlamydia* vaccine vectors. 

To improve the protective efficacy of this ECG-based vaccine against *C. abortus* infection, we used CTB to modify the rECG and evaluated its immune effects. The results showed that MIP antigens, fused or not fused to CTB, can be successfully expressed in ECGs and that the expression level accounts for approximately 1.2% of the total ghost protein. This percentage was significantly higher than that previously achieved when InpN was used as the leader sequence (approximately 0.28%) [8]. The fusion MIP could be delivered to the surfaces of the ECGs through the lpp’-ompA chimera. This chimera consists of the N-terminal signal peptide, the first nine residues of lpp and ompA responsible for targeting the outer membrane [15], and it is widely used because of its ability to carry large-molecular-weight proteins, its stability, and its high efficiency. The high expression of the MIP antigen may be related to the solubilizing effect of the N-terminal signal peptide in the lpp’-ompA chimera.

Previous studies have confirmed that BGs can improve the capture efficiency of DCs and stimulate their activation in vitro [26]. Moreover, displaying flagellin on the surfaces of BGs can enhance their ability to activate DCs and the immune efficacy of vaccines via the Toll-like receptor 5 pathway [27]. We observed that incorporating CTB significantly enhanced the activation ability of BMDCs in the rECG. From an immunological perspective, the incorporation of this adjuvant protein is beneficial. This is due to the adjuvant synergy between CTB and the pathogen-associated molecular patterns on the surfaces of rECGs. This synergistic effect activates the host’s innate immune system and induces a powerful adaptive immune response [28]. 

Numerous studies have shown that mucosal immunity is essential in protecting genital tract epithelial cells from *Chlamydia* infections. Moreover, the induction of Th1-type cellular immune responses is the key to successfully resisting *Chlamydia* infection [12]. Therefore, selecting appropriate adjuvants or delivery systems capable of inducing high-level secretory IgA production and activating Th1-type cellular immune responses is a major goal. As expected, intranasal inoculation with rECGs induced a Th1-biased cellular immune response in mice, consistent with previous findings in *V. cholerae* ghosts [29]. Moreover, incorporating CTB caused significant serum and genital tract IgA antibodies to be induced in the rECG via nasal immunization. Typically, the IgA titers in vaginal wash samples are significantly lower than those in serum [30]. Surprisingly, however, in the present study, the secretory IgA titers in the vaginal washings were almost identical to those in the serum. CTB is a widely used mucosal adjuvant that can bind to ganglioside GM1 on the cell surface, facilitating the interaction of the connected antigen protein with the mucosa. Moreover, CTB enhances the efficiency of antigen presentation by DCs and other antigen-presenting cells [31]. Therefore, it appears that the mucosal adjuvant property of CTB is responsible for the powerful secretory IgA response.

Despite the many successes in *Chlamydia* vaccine research to date, a truly effective and reliable vaccine remains unavailable. This is because traditional vaccines are usually weakly immunogenic and do not elicit an adequate immune response. On the other hand, in the face of complex intracellular parasitic pathogens, such as *Chlamydia*, the unilateral induction of a cellular or humoral immune response is insufficient [32]. We demonstrated that the rECG vaccines were highly protective in a mouse infection model, shortening the shedding time of *C. abortus* in the vagina and reducing the pathological damage to the uterus compared with the rMIP vaccine. Notably, the CTB-modified rECG showed more prominent protective efficacy. This may be due to the efficient antigen delivery ability and powerful adjuvant properties of rECGs, which induce a strong and comprehensive immune response. 

## 5. Conclusions

This study revealed the feasibility of using the mucosal adjuvant CTB to modify rECGs and enhance their immune adjuvant properties. rECGs that were surface infiltrated into CTB induced intense mucosal (IgA) and systemic (IgG, IgG1, and IgG2a) antibody responses, significantly induced a Th1-biased cellular immune response, and further reduced *C. abortus* infection and pathological damage. Therefore, rECG-lpp’-MIP-CTB represents a safe and effective subunit vector vaccine against *C. abortus*. Furthermore, this CTB-mediated approach expands the application of BG-based adjuvant-free vaccine platforms.

## Figures and Tables

**Table 1:** Primers used in this study.

Primer Name	Primer Sequence (5′–3′)	Restriction Site
MIP-F	ACT *GGATCC* GATCAGAGCAGCCATAACGA	BamH I
MIP-R	ATT *GTCGAC* GCTCGCGGTGTTTTTATCT	Sal I
lpp-F	ACT *CCATGG* AAGCTACTAAACTGGTA	Nco I
lpp-R	ACT *GGATCC* CTTGTCATCGTCGTCCTTGTA	BamH I
CTB-F	ATA *GTCGAC* ACCCCGCAGAATATCAC	Sal I
CTB-R	ATT *GCGGCCGC* TTAGTTCGCCATGCTAAT	Not I

Note: italics indicate restriction enzyme sites.

**Table 2:** MIP expression levels in *E. coli* strains.

Vaccine	MIP (μg/mL)	Total Protein (μg/mL)	MIP/Total Protein (μg/mg)
rECG-lpp’-MIP	61.49	5037.88	12.21
rECG-lpp’-MIP-CTB	60.69	5108.64	12.28

## Data Availability

All relevant data are included within the paper.
